# Original Communications

**Published:** 1873-02

**Authors:** 


***Original Communications***

Are solicited from our professional friends, but we must decline to insert them as such when previously sent to other medical journals, or especially to non-professional periodicals.

☞ The Annual Commencement of Rush Medical College will take place at Central Hall, corner of Wabash avenue and Twenty-second street, on Wednesday, February 19th, at 8 P.M. The Valedictory Address will be delivered by Prof. Walter Hay.

